# Water-Gated Transistor Using Ion Exchange Resin for Potentiometric Fluoride Sensing

**DOI:** 10.3390/mi11100923

**Published:** 2020-10-05

**Authors:** Zahrah Alqahtani, Nawal Alghamdi, Thomas J. Robshaw, Robert Dawson, Mark D. Ogden, Alastair Buckely, Martin Grell

**Affiliations:** 1 Physics and Astronomy, The University of Sheffield, Hicks Building, Hounsfield Rd, Sheffield S3 7RH, UK; nhalghamdi1@sheffield.ac.uk (N.A.); alastair.buckley@sheffield.ac.uk (A.B.); 2 Department of Physics, University of Taif, Taif-Al-Haweiah 21974, Saudi Arabia; 3 Department of Physics, University of Tabuk, King Fahad Road, Tabuk 47731, Saudi Arabia; 4 Department of Chemical and Biological Engineering, The University of Sheffield, Mappin St, Sheffield S1 3JD, UK; TJRobshaw1@sheffield.ac.uk (T.J.R.); m.d.ogden@sheffield.ac.uk (M.D.O.); 5 Department of Chemistry, The University of Sheffield, Dainton Building, Brook Hill, Sheffield S3 7HF, UK; r.dawson@sheffield.ac.uk; 6 Llyfrgell Bangor, Gwynedd Rd, Bangor LL57 1TD, UK; martin@spinne.plus.com

**Keywords:** Puromet, fluoride, sensor, WGTFT, ion exchange

## Abstract

We introduce fluoride-selective anion exchange resin sorbents as sensitisers into membranes for water-gated field effect transistors (WGTFTs). Sorbents were prepared via metal (La or Al)-loading of a commercial macroporous aminophosphonic acid resin, Puromet^TM^ MTS9501, and were filled into a plasticised poly(vinyl chloride) (PVC) phase transfer membrane. We found a potentiometric response (membrane potential leading to WGTFT threshold shift) to fluoride following a Langmuir–Freundlich (LF) adsorption isotherm with saturated membrane potential up to ~480 mV, extremely low characteristic concentration c_1/2_ = 1/K, and picomolar limit of detection (LoD), even though ion exchange did not build up charge on the resin. La-loading gave a superior response compared to Al-loading. Membrane potential characteristics were distinctly different from charge accumulating sensitisers (e.g., organic macrocycles) but similar to the Cs^+^ (cation) selective ion-exchanging zeolite mineral ‘mordenite’. We propose a mechanism for the observed threshold shift and investigate interference from co-solutes. Strong interference from carbonate was brought under control by ‘diluting’ metal loading in the resin. This work sets a template for future studies using an entirely new ‘family’ of sensitisers in applications where very low limit of detection is essential such as for ions of arsenic, mercury, copper, palladium, and gold.

## 1. Introduction

In recent years, water-gated thin-film transistors (WGTFTs) have been developed into a novel potentiometric transducer for the sensing of waterborne ions. WGTFTs were sensitised with plasticised poly(vinyl chloride) (PVC) phase transfer membranes loaded with ion-selective sorbents such as organic macrocycles [1,2,3]. Macrocycles selectively ‘accumulate’ ions (usually cations) in their central cavity. Consequently, they build up charge and potential at the membrane/water interface. The potential is quantitatively described by a Nikolsky–Eisenman law, which is a modification of the generic Nernst law. The Nernst law is logarithmic with ion concentration, c, without lower cut-off. This leads to a formal divergence in the limit c → 0 that is not practically observed. The Nikolsky–Eisenman law resolves this by introducing a lower ‘cut-off’ at a concentration called ‘c_st_’ in some previous literature, e.g., [3] and references therein. Below c_st_ the Nikolsky–Eisenman response flatlines. Therefore, c_st_ sets a limit of detection (LoD), and typical values for c_st_ are a few 100 nm, e.g., [1,2,3]. A similar characteristic applies to the classic potentiometric sensing of waterborne fluoride anions: Frant and Ross [4] reported electrodes constructed from LaF_3_ giving a Nikolsky–Eisenman characteristic with c_st_~1 µm and good selectivity for fluoride over other common anions. Alternatively, we have recently demonstrated WGTFT-based potentiometry using an ion exchange sorbent in the phase transfer membrane—the caesium-selective zeolite ‘mordenite’ [5], and the lead and copper-selective zeolite ‘clinoptilolite’ [6]. We found a membrane potential in response to increasing analyte concentration following a Langmuir or Langmuir–Freundlich isotherm (to be introduced below), instead of a Nikolsky–Eisenman law. The different response characteristics allow a much lower limit of detection. Moreover, the response characteristics of a catalyst-sensitised WGTFT for the non-ionic water pollutant benzyl alcohol also followed a Langmuir isotherm [7]. The origin of the membrane potential remained unclear, however: ion exchange does not accumulate a net charge on the membrane. This sets such membranes apart from membranes that are sensitised (e.g., with ion-selective organic macrocycles [1,2,3]), which give a Nikolsky–Eisenman membrane potential as a result of charge accumulation when exposed to a ‘target’ ion.

Here, we targeted the fluoride anion with another ion exchange sorbent in a WGTFT membrane. Fluoride is naturally present in groundwater. Small quantities of fluoride are beneficial to health, but excessive fluoride intake can result in fluorosis [8,9], a serious illness. The World Health Organization (WHO) sets a maximum acceptable concentration (‘potability limit’) of 1.5 mg L^−1^ = 79 μM fluoride in drinking water [9]. The health hazards associated with fluoride call for both methods of defluoridation and sensor technologies to detect fluoride hazards. Precipitation defluoridation results in large volumes of low-value slurry from which fluoride recovery is difficult [10]. Adsorption and ion-exchange techniques allow selective fluoride removal under mild conditions without generating waste. Numerous fluoride uptake studies have used sorbents such as alumina [11], activated carbon [12], chitosan [13], synthesised microporous polymers [14], and layered double hydroxide clays [15].

Recently, sorbents have been developed from the microporous Puromet^TM^ MTS9501 chelating resin that allows metal loading through its aminophosphonic acid group. Aluminium (Al)-loaded chelating resins have been investigated for fluoride-removal capabilities [16] and are commercialised for industrial use [17]. We successfully trialled lanthanum (La) loading [18]. Generally, sorbate extraction is quantified by relative sorbent mass uptake vs. sorbate concentration in solution, Equation (1):

(1) $$\frac{∆m\left(c\right)}{m\left(0\right)}=\frac{∆m\left(\infty \right)}{m\left(0\right)}\;\theta \left(c\right)$$

wherein *c* is the sorbate’s concentration, $\frac{\Delta m\left(\infty \right)}{m\left(0\right)}$ the sorbent’s capacity, i.e., saturated relative mass uptake, and θ(*c*) a dimensionless monotonously increasing function with θ(0) = 0 and θ(*c* → ∞) = 1. Note that the adsorption is defined in terms of *c* = *c*_f_, the final equilibrium concentration of sorbate in the solution after partial extraction by the sorbent, not c_i_, the initial concentration of sorbate before contact with sorbent. An often used, versatile approach to θ(*c*) is the Langmuir–Freundlich (LF) isotherm:

(2) $$\theta \left(c\right)={\left(Kc\right)}^{\beta }/[{\left(Kc\right)}^{\beta }+1]$$

which generalises the classic Langmuir isotherm (special case *β* = 1). *K* quantifies the strength of the interaction between a sorption site and the sorbate, *β* ≤ 1, quantifies inhomogeneity between sorption sites (*β* = 1, *K* for all sorption sites is equal). A characteristic concentration c_1/2_ is given by θ (c_1/2_) = ½. For the LF isotherm, c_1/2_ = 1/*K*, but c_1/2_ can be read directly from measured characteristics without relying on a model. Note, for mordenite extracting Cs^+^, c_1/2_ is very much smaller for membrane potential [5] than for mass extraction [19].

**Figure 1:**
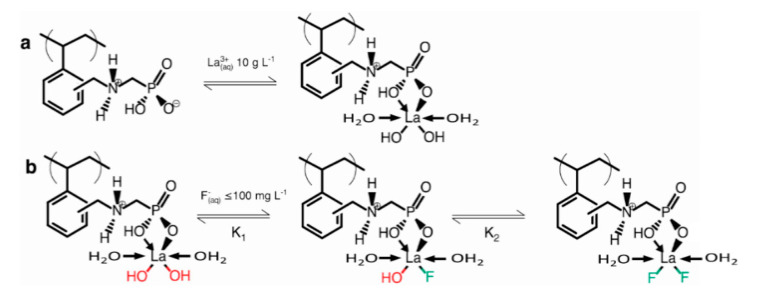
Sorbent resin chemistry. (**a**) Activation of Puromet^TM^ MTS9501 resin with trivalent metal (for example, La) for later fluoride uptake. (**b**) Ligand exchange (F^−^ for OH^−^) when La-activated Puromet^TM^ comes in contact with aqueous F^−^ (e.g., from NaF) based on [18]. Note: The full La inner coordination sphere is not shown for reasons of space.

Here, we introduced metal-loaded (La and Al) Puromet ion-exchange chelating resins into WGTFTs, the first example of an anion (fluoride)-selective potentiometric WGTFT sensor. Chemically, the amorphous organic resin (cf. Figure 1a) is very different to inorganic crystalline zeolites such as ‘mordenite’ and ‘clinoptilolite’ zeolite minerals we used previously for Cs^+^ [5] and Pb^2+^/Cu^2+^ [6] sensing respectively. However, they both exchange rather than accumulate ions, and we again found a response characteristic controlled by Equation (2) with very low c_1/2_ and LoD. We investigated the mechanism behind the build-up of an ion concentration-dependent potential in phase transfer membranes sensitised with ion exchange sorbents despite the lack of a net charge on the sorbent and tested a proposed model. Furthermore, we explored practical aspects (e.g., interference from other anions), and the use of WGTFTs to predict extraction efficiency on very small sorbent samples. Most importantly, our work sets a template for the use of a new family of sensitisers, namely ion exchange resins, in WGTFT-based sensors for water pollutants. Such resins are available as sorbents for very harmful, or precious, water pollutants (arsenic, gold, copper, mercury, palladium [20,21,22,23,24]). Following this work, these sorbents can now easily be adapted for the sensing of such pollutants as well.

## 2. Experimental Procedure and Evaluation

### 2.1. Preparation of Metal-Loaded Chelating Resin

Puromet^TM^ MTS9501 [25] is a macroporous, weakly acidic chelating resin, consisting of a styrene/divinylbenzene cross-linked copolymer backbone with pendant aminomethylphosphonic acid (AMP) functional groups that allow metal loading. It is delivered in the form of microspheres ~300 μm in diameter. The La loading is described in [18], and for Al loading in [16]. References [16] and [18] also give details on the reagents used for the loading procedures. Metal loading is illustrated in Figure 1a. Briefly, trivalent metal cations are bonded via chelation interaction with the AMP group whilst retaining a number of inner coordination sphere sites, which are occupied by hydroxyl and water moieties. Metal loading activates the resin for fluoride uptake via ligand exchange at low concentrations by replacing one or both hydroxyl (-OH) ligands in R-M(-OH)_2_ (M = La or Al) with waterborne fluoride, F^−^_aq_ (illustrated in Figure 1b). We note that both the La-OH bond and the La-F bond are polar and thus contribute a dipole moment, but these will not be of the same magnitude. Hence, ion exchange of F^−^ for OH^−^ changes dipole moment, which in principle can be detected with a potentiometric transducer. The characteristic concentration, c_1/2_, for the extraction of fluoride with Al-loaded Puromet^TM^ MTS9501 chelating resin was c_1/2_ = 370 μm [16] while La-loaded chelating resin gave lower c_1/2_ = 160 μm [18]. The lower c_1/2_ for La—vs. Al-loaded resin is a quantitative measure for the stronger fluoride sorption by the La-loaded resin. Note the sorbent’s capacity $\frac{\Delta m\left(\infty \right)}{m\left(0\right)}$ may nevertheless be larger for Al-loading as this is controlled by the extent of metal loading, not the strength of sorption. To prepare the resin for incorporation into the WGTFT, it was dried in a 50 °C air-flow oven for 24 h, then ground to various degrees with pestle and mortar.

The La-loading procedure described in [18] results in ‘heavily’ La-loaded resins carrying 256 ± 2 mg of La per gram of resin. To prepare resins that are only ‘lightly’ loaded with La (to be used in Section 3.4), a heavily loaded resin sample (25 g hydrated mass) was placed in a polypropylene bottle with 1 L of 1 M HCl. This was sealed and placed on an orbital shaker for a period of 24 h, after which the resin was separated from the acidic solution, placed under gravity filtration and washed with at least 5 L of deionised water until the pH of the filtrate was near neutral.

### 2.2. Preparation of Ion-Selective Membranes

**Figure 2:**
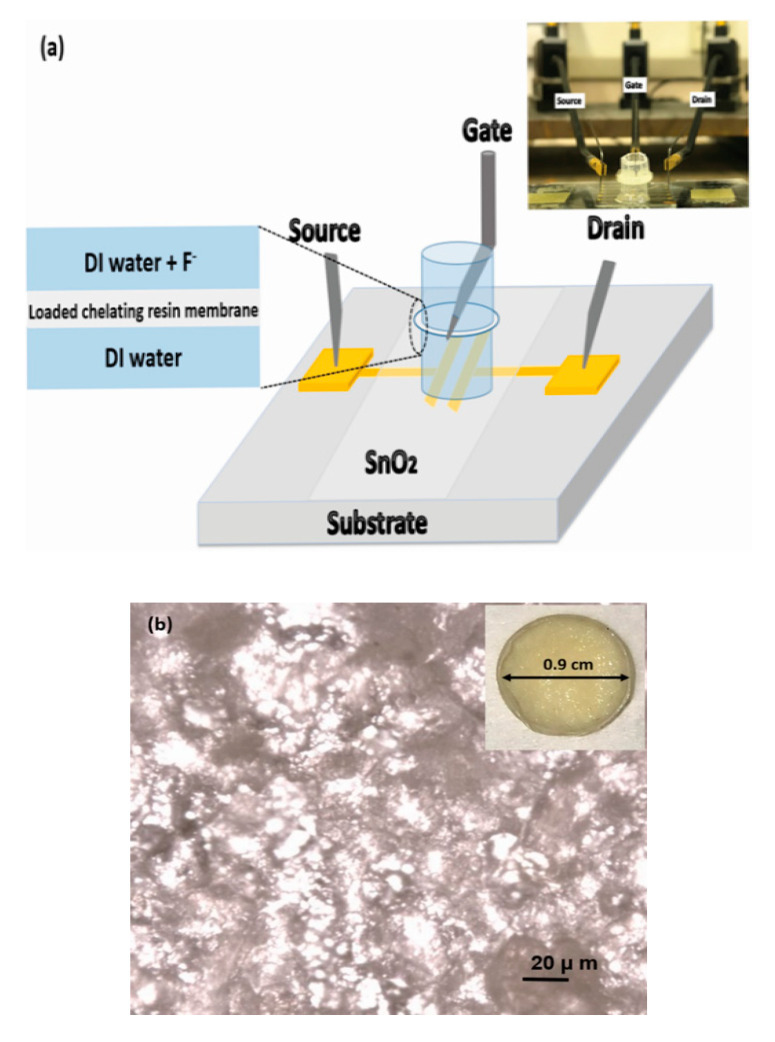
Measurement setup and membrane morphology. (**a**) Water-gated thin-film transistors (WGTFT) setup for fluoride sensing. Inset to 2a: photograph of the chelating resin-sensitised SnO_2_ WGTFT sensor platform. (**b**) Micrograph of La-loaded chelating resin distributed in poly(vinyl chloride) (PVC) phase transfer membrane, in which the darker section of the image is for resin while lighter is for PVC. Inset to 2b: Photograph illustrates the diameter of La- chelating resin-loaded PVC phase transfer membrane. Resin loading in the membrane was 5.6 mg.

Metal-loaded chelating resins were incorporated into plasticised PVC phase transfer membranes by first mixing 30 mg PVC, 63 µL 2-nitrophenyl octyl ether (2NPOE) as a plasticiser, 6 mg lipophilic additive potassium tetrakis(4-chlorophenyl)borate (KTpClPB), and 39 mg metal-loaded chelating resin powder in 3 mL tetrahydrofuran (THF). PVC, 2NPOE (99%), KTpClPB, and THF (≥99.9%) were obtained from Sigma Aldrich (Gillingham, UK). While all the other components dissolved in THF, the resin dispersed as a suspension. 450 µL of membrane mixture solution was placed in a small vial and left overnight to allow solvent evaporation, resulting in membranes carrying 5.6 mg metal-loaded chelating resin each. The resulting membranes (micrograph shown Figure 2b) were ~0.4 mm thick and were then conditioned in deionised (DI) water for one day. The membrane was later introduced into a water-gated thin film transistor in between two plastic pools with epoxy glue as shown in Figure 2a.

### 2.3. Fluoride-Sensitive Water-Gated Thin Film Transistor (WGTFT) Setup

We used source/drain (S/D) gold/chrome adhesion-layer contact substrates with channel geometry width/length = 1 mm/30 µm connected by a semiconducting film of electron-transporting tin dioxide (SnO_2_) spray-pyrolysed from tin chloride solution, as described previously in [5]. Membranes, as described in Section 2.2, were glued between two pools to separate them into two compartments. The lower ‘reference’ pool was filled with DI water and was in contact with the SnO_2_ film at the bottom and the central membrane at its top. We filled the reference pool with DI water. This is justified for a LF response characteristic Equation (2), which gives membrane potential → 0 for c → 0. Note this is different from the conventional Nernstian/Nikolsky–Eisenman characteristics which imply non-zero potential for c → 0. Reference compartments for conventional potentiometric ion sensors therefore typically contain analyte at c >> c_st_ (e.g., [1]), but this is not indicated under the LF law. The upper ‘sample’ pool was filled with samples containing standard amounts of fluoride (from NaF or KF) or interferant (chloride or carbonate salts) dissolved in DI water, and was in contact with the central membrane at its bottom and a tungsten electrode at the top, which served as the transistor’s gate electrode. The setup is illustrated in Figure 2a. More details on the construction of the sample pools and the electric characterisation setup are given in [5] and references therein. After each measurement cycle, the sample pool was then emptied and re-filled with a different sample, usually with increasing salt concentration. Note that drinking water commonly contains further anions (e.g., chloride, nitrate, and carbonate) that are far more concentrated than fluoride and can be tolerated up to millimolar potability limits, as detailed in [26,27]. We have, therefore, also conducted experiments to study interference from such ions in a similar manner as described above for fluoride.

### 2.4. Determination and Evaluation of Membrane Potential with WGTFT

A voltage applied to the gate contact in Figure 2 is communicated across the water in both pools and the membrane to the semiconductor surface via interfacial electric double layers (EDLs). The membrane builds up a potential in response to different ion concentrations in the upper (sample) vs. the lower (reference) pool. This concentration-dependent membrane potential, *V_M_*(*c*), is added to the voltage applied to the gate. Thus, the potential at the semiconductor surface is different from the potential applied to the gate by *V_M_*(*c*). Consequently, the ‘threshold’ voltage *V_th_*, i.e., the gate voltage at which the transistor turns from ‘off’ to ‘on’ shifts by V_M_. We increased analyte concentration in the sample pool stepwise and allowed 6 min for the membrane to equilibrate. Then we recorded linear transfer characteristics similar as in [5]: At a small fixed positive source-drain voltage V_D_ (source on ground, drain on + 0.1 V), we swept the gate voltage V_G_ from a ‘large’ negative value (meaning, a value well below threshold, V_th_, where the transistor clearly is ‘off’) to a ‘large’ positive value, where the drain current I_D_ increases linearly with gate voltage, while measuring and recording I_D_. Note, due to the high capacitance of the EDL, a few 100 mV are sufficiently ‘large’ here. The resulting characteristic I_D_(V_G_) is known as ‘linear transfer characteristic’ due to the linear I_D_ vs V_G_ relation at high V_G_. Evaluation of linear transfers allows tracking *V_th_*, which under increasing analyte concentration in the sample pool, shifted according to the membrane potential, Δ*V_th_*(*c*) = *V_M_*(*c*). Therefore, we shifted measured characteristics graphically along the gate voltage (V_G_) axis until they best matched the zero fluoride (DI water) transfer, constructing a ‘master’ transfer characteristic. This method was first reported by Casalini et al., [28]. The shift needed to best match the characteristic under concentration, *c*, to the *c* = 0 characteristic, was identified as Δ*V_th_*(*c*) = *V_M_*(*c*). The shift procedure, including evaluation of errors, is illustrated in one example in Supplementary Material, Figure S6. Finally, we plotted the Δ*V_th_*(*c*) response, including error bars, and fitted to a Langmuir–Freundlich model, similar to that of sorbate mass uptake, using the non-linear fit routine in Origin 2018:

(3) $$V_{M}\left(c\right)=∆V_{th}\left(c\right)=∆V_{th}\left(\mathrm{sat}\right)\;\theta \left(c\right)=∆V_{th}\left(\mathrm{sat}\right)\;{\left(Kc\right)}^{\beta }/\left[{\left(Kc\right)}^{\beta }+1\right]$$

To determine the *LoD*, we plotted the same data in linearised form, Δ*V_th_*(*c*) ((*Kc*)*^β^* + 1) vs. (*Kc*)*^β^*, and fitted a straight line of the form Δ*V_th_*(*c*) ((*Kc*)*^β^* + 1) = *m*(*Kc*)*^β^* + *b*, evaluating parameters m and *b* +/− Δ*b* by Origin 2018′s linear fitting routine. *b* was expected to overlap zero within +/− Δ*b*. *LoD* was calculated from the common ‘3 estimated standard errors’ criterion [29]:

(4) $${\left(Kc_{\mathrm{LoD}}\right)}^{\beta }=3∆b/m\;$$

## 3. Results and Discussion

This section is structured into 4 Sections (Section 3.1, Section 3.2, Section 3.3 and Section 3.4). In Part Section 3.1, we discuss our use of WGTFTs to establish membrane potential characteristics in response to fluoride concentration in water samples. The determined characteristics led us to a hypothesis for the underlying mechanism that we tested and confirmed, the results of which are presented in Section 3.2. In Section 3.3, we then address some of the practical issues for using chelating resins in WGTFT fluoride sensors; namely, recovery and interference. Finally, reducing the interference from carbonate by diluting metal loading in the resin is presented in Section 3.4.

### 3.1. Fluoride Response for WGTFTs Using La- and Al-Loaded Resin Membranes

**Figure 3:**
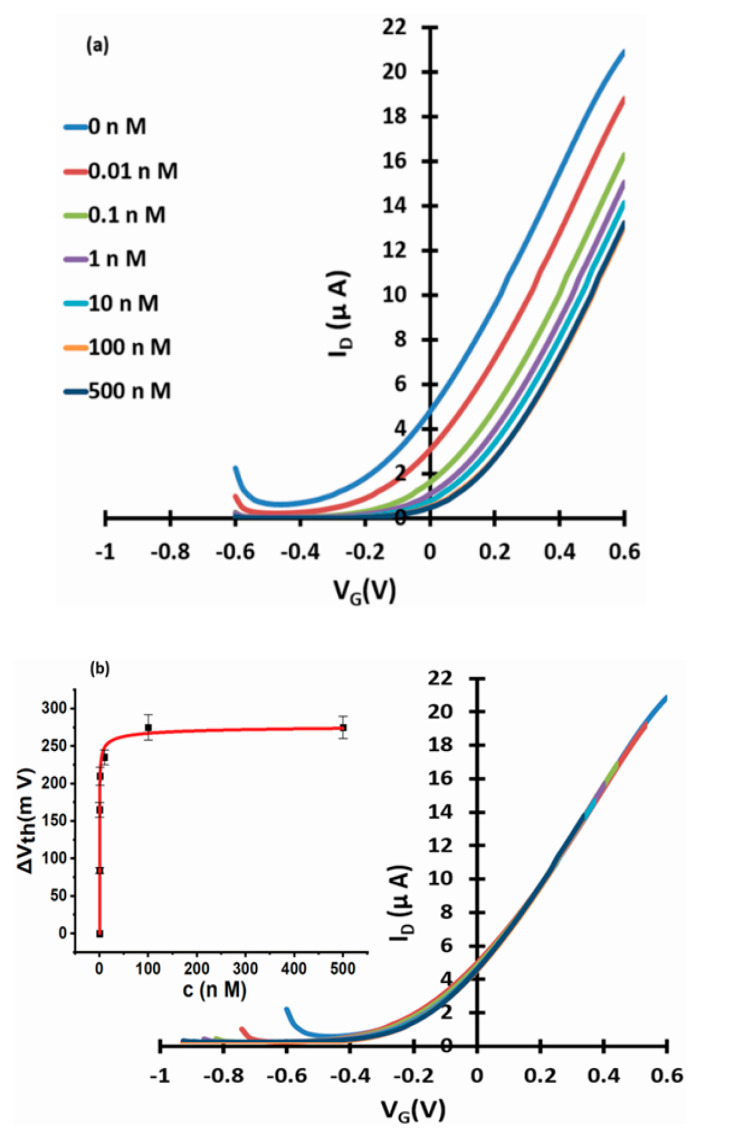
WGTFT fluoride (from NaF) response with La resin membrane. (**a**) Transfer characteristics of La-loaded chelating resin-sensitised SnO_2_ WGTFT gated under increasing F^−^ concentrations from NaF in the outer pool. (**b**) ‘Master’ transfer characteristic after shifting transfers from Figure 3a along the V_G_ axis for optimal overlap. Inset to 3b: response characteristic with fit to Equation (3).

**Figure 4:**
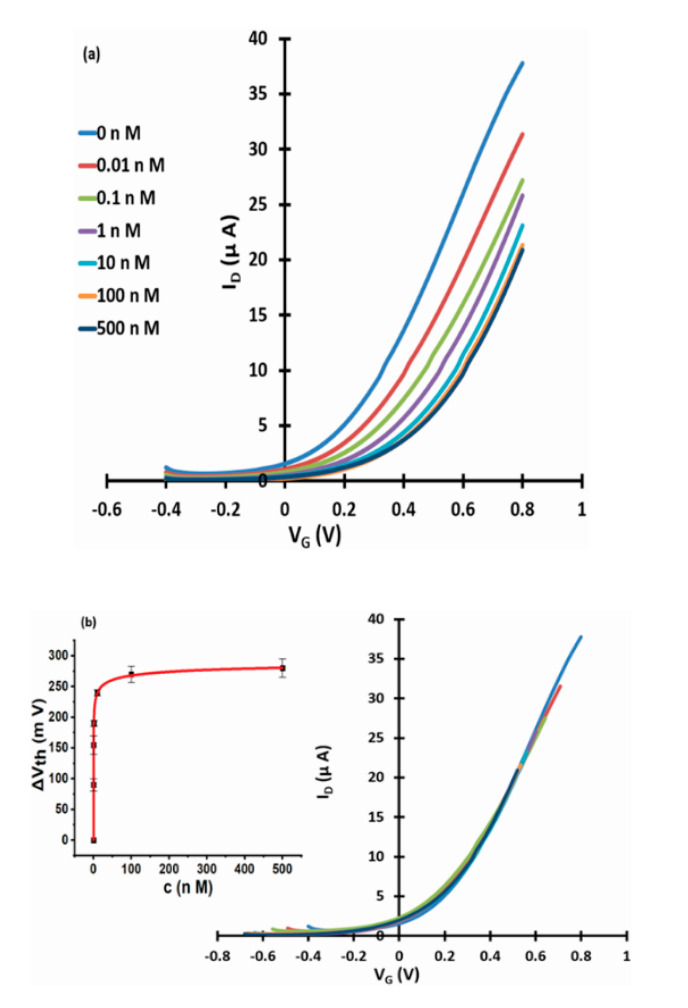
WGTFT fluoride (from KF) response with La resin membrane. (**a**) Transfer characteristics of La-loaded chelating resin-sensitised SnO_2_ WGTFT gated under increasing F^−^ concentrations from KF in the outer pool. (**b**) Master transfer characteristic. Inset to 4b: response characteristic with fit to Equation (3).

**Figure 5:**
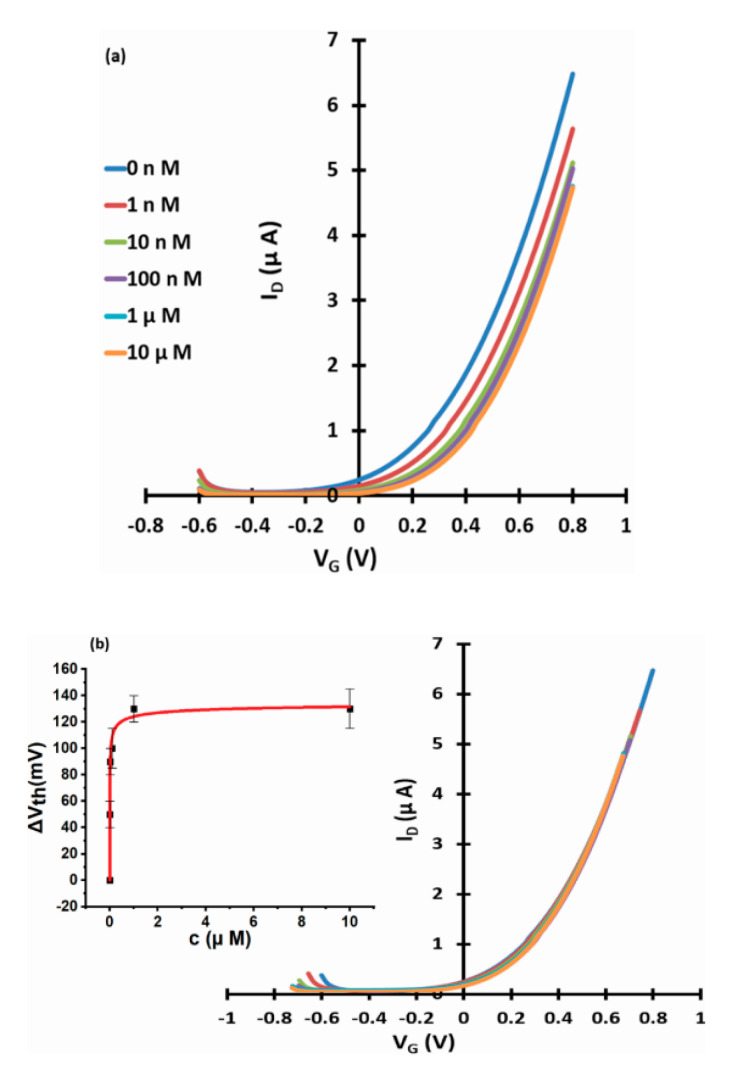
WGTFT fluoride (from NaF) response with Al resin membrane. (**a**) Transfer characteristics of Al-loaded chelating resin-sensitised SnO_2_ WGTFT gated under increasing F^−^ concentrations from NaF in the outer pool. (**b**) Master transfer characteristic. Inset to 5b: response characteristic with fit to Equation (3).

Figure 3, Figure 4 and Figure 5 show linear transfer characteristics of phase transfer membrane-sensitised WGTFTs under increasing concentrations of fluoride (from NaF or KF) in the upper (sample) pool, using La-loaded (Figure 3 and Figure 4) and Al-loaded (Figure 5) chelating resin membrane.

For both La- and Al-loaded membranes, transfer characteristics clearly shifted to more positive gate voltages under increasing fluoride concentration. For the La resin, this trend was already observed for a fluoride concentration of only 10 pM. This showed that metal-loaded membranes developed a potential in response to very small fluoride concentrations in the sample under test. Threshold shift was evaluated quantitatively, as described in Section 2.4. The resulting response characteristics for both La- and Al-loaded resin are shown as insets to Figure 3b, Figure 4b and Figure 5b, respectively, including fits to the LF model Equation (3). The data line up on a smooth curve fitted well by the LF law, without random scatter. This is empirical confirmation that the use of DI water in the reference pool here did not lead to unstable reference potentials. Table 1 summarises the parameters K, c_1/2_ = 1/K, ΔV_th_(sat), and β for the best fit to Equation (3), as well as LoDs. Evaluation of LoD was as described in 2.4. and is shown in the Supplementary Materials, Figure S1.

**Figure 6:**
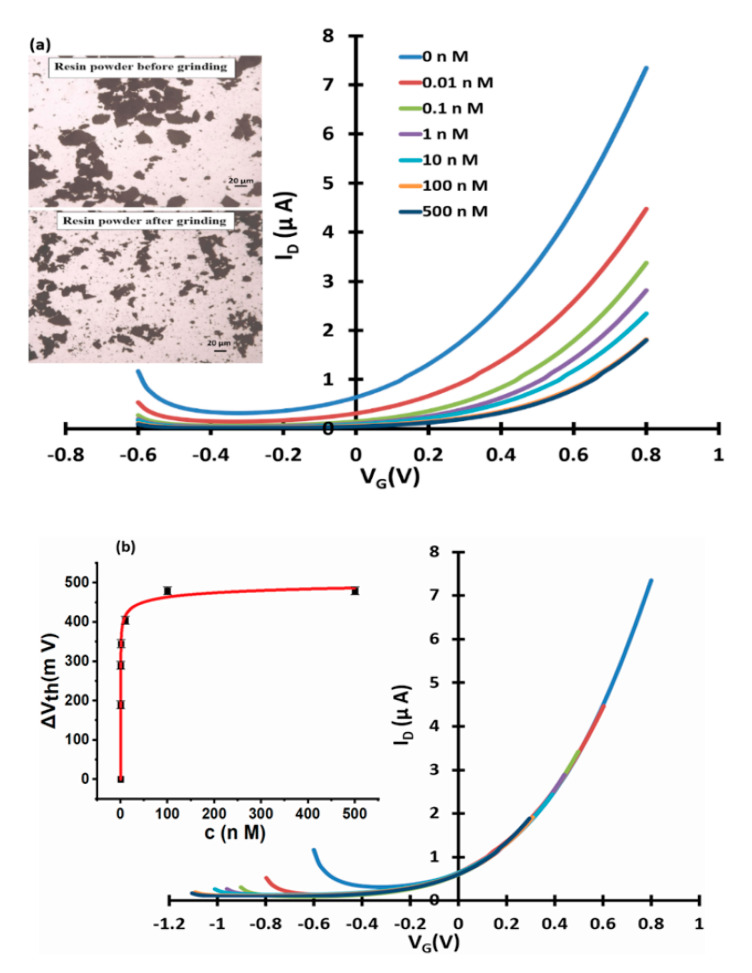
WGTFT fluoride response with finely ground La resin membrane. (**a**) Transfer characteristics of finer-ground La-loaded chelating resin-sensitised SnO_2_ WGTFT gated under increasing F^−^ concentrations from NaF in the outer pool. Inset to 6a: resin powder before/after grinding with pestle and mortar. (**b**) Master transfer characteristic. Inset to 6b: response characteristic with fit to Equation (3).

To ensure repeatability, we repeated recording transfer characteristics for La (coarse and fine powder, details in Section 3.2) and Al-loaded chelating resin-sensitised SnO_2_ WGTFT with increasing F^−^ concentrations from NaF in the outer pool with nominally identical devices and membranes. Results are shown as Supplementary Information. Figures S2 and S3 show coarse and fine powder La resin, respectively, and S4 shows coarse Al resin, corresponding to Figure 3, Figure 6, and Figure 5 in the original manuscript. The ΔV_th_ vs. fluoride concentrations resulting from repeat experiments are included with different symbols in the response characteristics (insets in Figures S2–S4). The parameters (K, c_1/2_, ΔV_th_(sat), β, and LoD) are summarised in Table S1, overlapping with Table 1 within the margin of error. This confirms repeatability.

We note and discuss a number of interesting properties of the observed response characteristics. Threshold shifts (i.e., membrane potentials) are fitted well by a characteristic of the form Equation (3), which is based on a Langmuir–Freundlich (LF) isotherm, albeit errors in some parameters are relatively large. This is common though for multiparametric non-linear fits [30,31]. The response following LF characteristics is distinctly different from the Nikolsky–Eisenman (Nernstian with lower limit ≈ LoD) characteristics typically observed for potentiometric sensors, including (cat)ion-selective WGTFTs sensitised with organic macrocycles (e.g., [1,2,3]), and LaF_3_ membrane-based fluoride potentiometers [4]. However, we have recently reported membrane potential and threshold shift with Langmuir characteristics [5] and LF characteristics [6] for a cation-selective WGTFT sensitised with zeolites. We suggest this difference is rooted in different sorption mechanisms: When an organic macrocycle complexes an ion (usually a cation) in its central cavity, it does so without ion exchange. Hence, the membrane usually accumulates positive charge, leaving behind an excess of negative charge in the aqueous phase. Consequently, an electric double layer (EDL) forms at the membrane/water interface, with associated membrane potential. The quantitative treatment of ion complexation from solutions of target ions with concentration c leads to the Nernst equation, with a logarithmic dependence of membrane potential on c. However, chelating resins and zeolite extract sorbate ions by ion exchange without build-up of net charge in the sorbent: e.g., La-activated Puromet resin returns a hydroxyl (OH^−^) ion to the aqueous phase for every fluoride (F^−^) ion it extracts from it (Figure 1b). Neither the membrane nor aqueous phase accumulated net charge; hence, the assumptions of the Nernst law are not given. Ion exchange rather followed an LF adsorption isotherm Equation (2). How ion exchange can nevertheless lead to a membrane potential will be discussed below. First, we note a striking quantitative difference between the K’s/c_1/2_’s for sensing (i.e., threshold shift) and extraction (i.e., mass uptake) in ion exchange sorbents: for La-loaded resin, Table 1 shows c_1/2_ for threshold shift is 6½ orders of magnitude smaller (K correspondingly larger) than for mass extraction with the same sorbent [18]. K for threshold shift is also very large in comparison to K’s found for the binding between metal cations and selective organic dyes (e.g., [29]), which are of a similar order (or somewhat larger) to K’s for mass extraction. We found a similar discrepancy in c_1/2_ for caesium-selective mordenite zeolite ion-exchange sorbent, c_1/2_ ≈ 260 pM for threshold shift [5] vs. c_1/2_ ≈ 640 μM for mass uptake [19]. For reasons not well understood at this stage, ΔV_th_ already saturates when only a small fraction of all available ion exchange sites have exchanged hydroxyl for fluoride. The very small c_1/2_ moderates the caveat we gave in the introduction on the difference between the initial concentration, c_i_, and c_f_, the final concentration of sorbate (e.g., fluoride) after contact with sorbent (e.g., chelating resin) in the extraction characteristics Equation (1).

To address the fundamental question of how a membrane potential develops when neither membrane nor aqueous phase acquire a net charge under ion exchange, we note that the R–La–OH and the R–La–F bonds have a different dipole moment. Hence ion exchange R–La–OH + F^−^ → R–La–F + OH^−^ leads to a change in the magnitude of the dipole moment at the exchanged site. The density of dipole moments represents a polarisation, P. We believe it is this polarisation, in particular at the surfaces of resin grains, that leads to a shift in threshold voltage. It is well established that polarisation can gate field effect transistors, the most prominent example being memory transistors using ferroelectric or similar gate media (as in [32]). Such transistors can be ‘on’ even at zero applied gate voltage, solely due to the gate medium’s remnant polarisation. A surface mechanism is suggested by the results presented in Section 3.2 below.

While the discussion above applies to all ion exchangers, such as chelating resins and zeolites, we observed a minor difference in their response characteristics. While the generic Langmuir isotherm (special case of LF with β = 1) provides a good fit for threshold shift in WGTFTs using zeolite ‘mordenite’ sorbent for Cs^+^ [5], threshold shift characteristics for zeolite ‘clinoptilolite’ under lead and copper [6], and for chelating resin under fluoride as reported here, show β values significantly smaller than 1. Langmuir isotherm theory assumes all sorption sites have equal constant K, and β < 1 in the LF isotherm accounts for dissimilar K within the same sorbent. Thus, the more inhomogeneous sorption sites there are, the smaller β becomes [33]. We note that while mordenite has a clearly defined chemical makeup as well as a defined crystalline unit cell, clinoptilolite has a degree of randomness in the cations available for ion exchange. The chemical composition of the clinoptilolite unit cell is given as (Na, K, Ca)_3–6_(Al_6_Si_30_O_72_).20H_2_O [34], indicating different abundance of Na^+^, K^+^, and Ca^2+^ cations for exchange within different unit cells (albeit always adding up to overall oxidation state +6). This will lead to a distribution of ion exchange enthalpies, and hence K’s. Similarly, our organic chelating resin is an amorphous material, therefore different exchange sites will experience different microenvironments. Additionally, in the macroporous environment, in some cases, two metal centers may be adjacent and ‘share’ a fluoride-bridging ligand [35]. Overall, this again leads to a distribution of ligand exchange enthalpies, and consequently, K’s.

Using a different salt (NaF vs. KF) to introduce fluoride did not lead to a significant difference in the response characteristic parameters within their errors. However, we observed a significant difference between La- and Al-loaded resins. The higher affinity of fluoride to exchange for OH^−^ from La-OH rather than an Al-OH, as also shown in the extraction characteristics presented in [18] and [16] is reflected in the lower c_1/2_ for membrane potential and higher saturated threshold shift ΔV_th_(sat) for La- rather than Al-loaded resin. While c_1/2_s are very different between mass extraction and membrane potential, they do scale in proportion.

ΔV_th_(sat) for La-loaded resin of ~300 mV stands tall within the ‘electrochemical window’ of water (1230 mV). Nernstian threshold shift is only 58 mV per decade in ion concentration. The remarkably large membrane potential under minute concentrations of fluoride also leads to extremely small LoDs, many order-of-magnitude below the potability limit, and below the LoD with LaF_3_-based potentiometry [4]. This justifies the study here of sensing against a background of deionised water rather than against typical drinking water, as for instance in [5,6]. While our local tap water typically contains fluoride well below potability (3 µM vs. 79 µM potability) [36], this still far exceeds c_1/2_ of our sensors and would push them into saturation.

### 3.2. Fluoride Response Using La-Loaded Resin of Different Grain Sizes

To explore the importance of resin grain surfaces to the build-up of membrane potential, we compared La-loaded chelating resin phase transfer membranes carrying the same weight of resin, but with different grain sizes. This was done by grinding the original coarse powder finer with a pestle and mortar before loading into the phase transfer membrane. As inset to Figure 6a, we show micrographs of resin grains before and after grinding, displaying a finer texture after grinding. The resulting membranes hence carried the same mass and volume of resin, but the finer ground powder led to a larger sorbent surface area. Figure 6 shows the response characteristics of a WGTFT that is otherwise nominally identical to the WGTFT used for Figure 3, but with finer ground resin in the phase transfer membrane. Note that a corresponding test was not possible for the ‘mordenite’ [5] and ‘clinoptilolite’ zeolites [6], as they were delivered as a very fine powder which we could not grind any finer with pestle and mortar.

The parameters of the fit of response characteristics to Equation (3) are included in Table 1 above in the ‘La (fine)’ row for direct comparison with ‘La (coarse)’ (i.e., parameters for the coarser powder). We found that within the margin of error, parameters K and β were not affected by grinding the powder finer. K describes the (average) strength of interaction between a single sorbent site and the sorbate, β, the inhomogeneity of such strengths in a disordered medium. Unsurprisingly, neither of those was affected by mechanical grinding that affects morphology on the size scale of µm. However, the saturated threshold shift, ΔV_th_(sat), was significantly larger for the finer ground powder with larger sorbent surface area. This suggests that membrane potential in ion exchange (rather than charge accumulating) sorbent membranes results from dipoles forming via sorption of sorbate on grain surfaces. Grinding ion-exchange media into finer powders allows the further increase of an already large ΔV_th_(sat), which benefits LoD, and as we will see in Section 3.3, the discrimination between analyte and interferant.

### 3.3. Recovery and Interference from Co-Solutes

**Figure 7:**
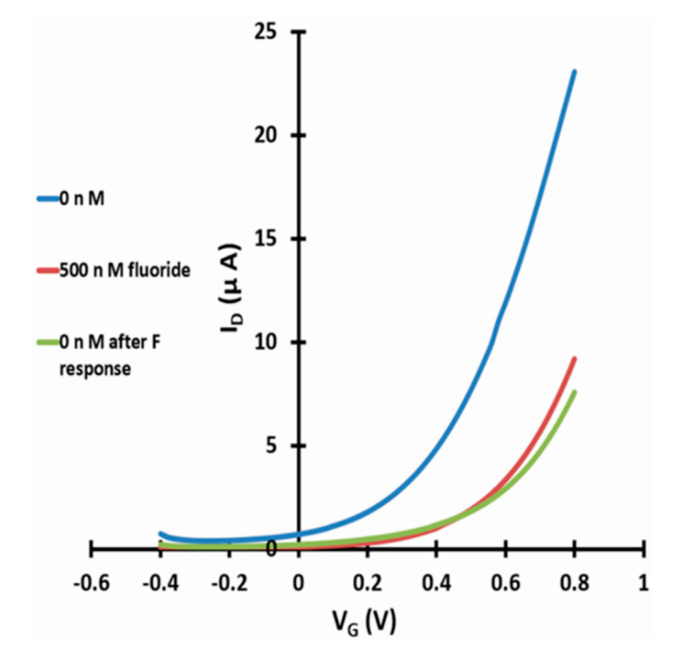
WGTFT fluoride response and recovery. Linear transfer characteristics for a WGTFT sensitised with La-loaded chelating resin under a test cycle of DI water (0 nm fluoride) ⇒ 500 nm fluoride ⇒ DI water (0 nm fluoride) again in the sample pool.

As c_1/2_ for fluoride response established in Section 3.1 is more than 6 orders-of-magnitude smaller than the potability limit of 79 µM, practical sensing would require manifold (factor~10^6^) dilution of test samples with DI water to bring natural fluoride concentrations into the sensor’s dynamic range (avoiding saturation). However, this is easily done. Note this would also dilute any co-ions present in realistic samples, justifying our choice to use DI water as reference. More important practical considerations are the ability of a sensor to recover, and the resilience against interferants (other anions and cations) in water samples that may also lead to a threshold shift. To test for recovery, Figure 7 shows linear transfers for a WGTFT sensitised with La-loaded chelating resin under a test cycle of DI water (0 nm fluoride) ⇒ 500 nm fluoride >> c1/2 ⇒ DI water (0 nM fluoride) again in the sample pool. It is evident from Figure 7 that a phase transfer membrane that was once exposed to a level of fluoride far larger than c_1/2_ does not recover zero membrane potential when the sample pool is re-filled with DI water, but remains at or near saturated threshold shift. This is likely due to the strong analyte/sorbent binding, as quantified by large binding constant, K. Note, water deioniser columns also do not easily recover after use when flushed with DI water. Recovery may be possible by prolonged washing under dilute NaOH to reverse the fluoride/hydroxyl ligand exchange, but we have not attempted this. Since each membrane carries only a few milligrams of La-loaded resin, it is cheap enough to discard after single use.

**Figure 8:**
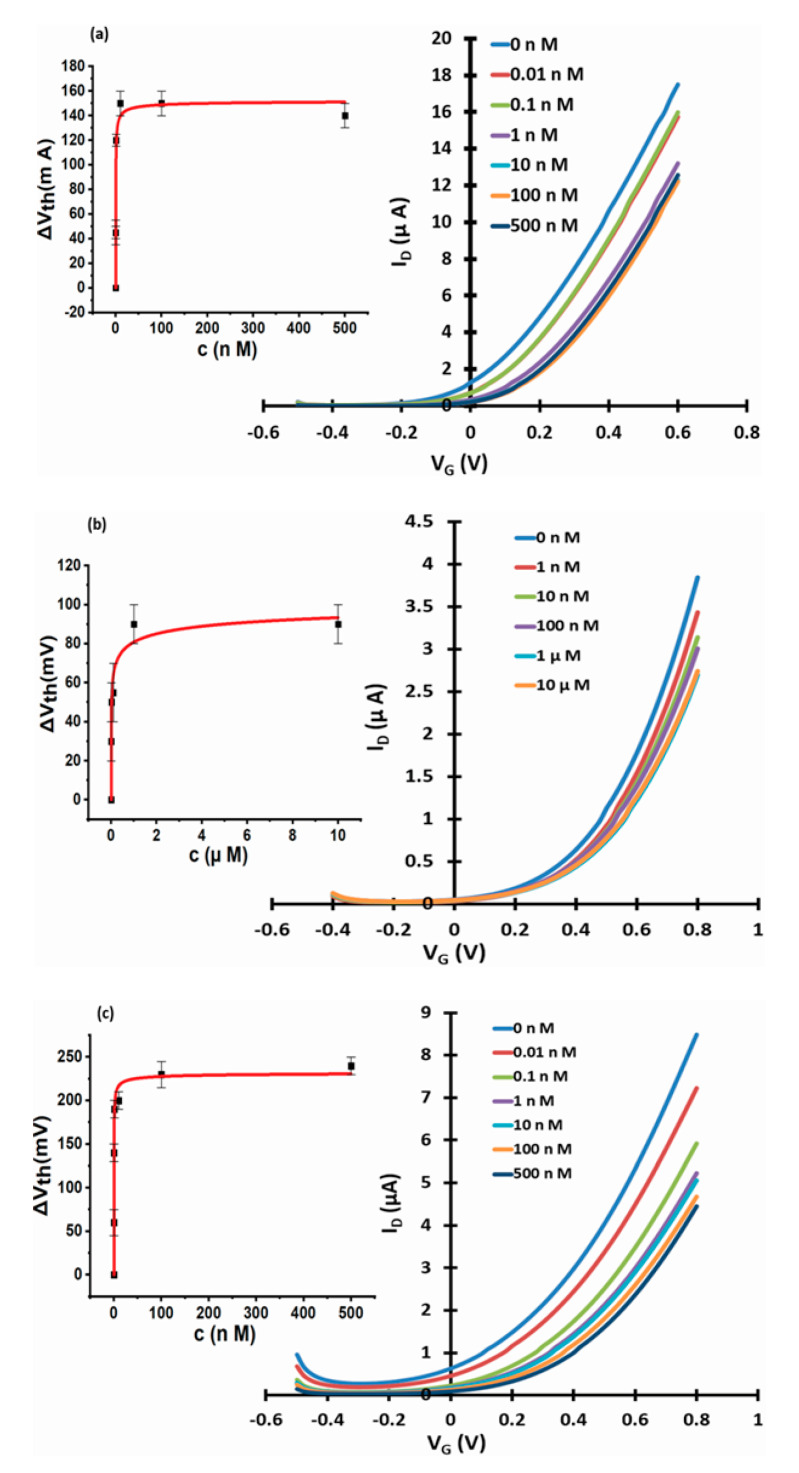
WGTFT fluoride sensors under chloride and carbonate as interferants. (**a**) Response of La-loaded resin to chloride from NaCl. (**b**) Response of Al resin to chloride from NaCl. (**c**) Response of La resin to carbonate. Insets: Response characteristics with fits to Equation (3).

To test for interference from other waterborne anions, we have measured the response of WGTFTs sensitised with La-and Al-loaded chelating resin to chloride (from NaCl), and for La-loaded phase transfer membrane to carbonate (from Na_2_CO_3_) in the same manner as done previously for fluoride. Chloride (Cl^−^) is the most common monovalent anion in drinking water and is typically far more concentrated than fluoride: The potability limit for chloride is 7 mM [26], almost 2 orders-of-magnitude larger than for fluoride. Carbonate (CO_3_^2−^) is a common divalent anion, and the recommended range for drinking water is (0.3 … 4) mM [27], around one order-of-magnitude larger than for fluoride. Note that although we introduced this as a divalent carbonate anion from Na_2_CO_3_ at low concentration (i.e., ’*mild’ pH*), most ‘carbonate’ ions will in fact convert to the monovalent bicarbonate, HCO_3_^−^, rather than solvate as true carbonate [37]. Results are presented in Figure 8 below, clearly showing non-negligible membrane potential in response to chloride and carbonate.

Transfers were again shifted for best overlap into a master curve (not shown here), and threshold shift characteristics are shown as insets with fits to Equation (3). We found that for chloride, the LF isotherm model Equation (3) does not fit the data as well as previously in Figure 3 and Figure 4. Therefore, some of the resulting ‘LF’ parameters carry large errors (particularly, K), and should be treated with caution. We still summarise them in Table 2, but prefer reading c_1/2_ directly from response characteristics, without reliance on the LF (or any other) isotherm model.

**Figure 9:**
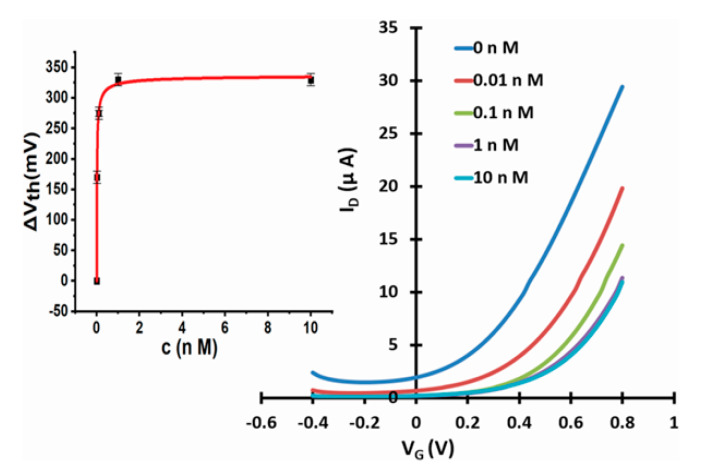
WGTFT fluoride sensors under simultaneous analyte and interferant. Response characteristics under simultaneous exposure to fluoride and chloride. The given concentrations apply to both fluoride and chloride: 1 nm means ‘1 nM fluoride + 1 nm chloride’. Inset: Response characteristics with fit to Equation (3).

Again, response to chloride was stronger for La- vs. Al-loaded resin. For both Al- and La-loaded resins, c_1/2_ is larger for chloride than for fluoride, but only by one order-of-magnitude. Since the typical concentration of chloride in common tap water is larger than fluoride, chloride could therefore still be a problematic interferant for the determination of fluoride. However, chloride ions are known not to act as inner-sphere ligands for La^3+^ aqueous complexes, [38], suggesting a much smaller change in dipole moment for La–OH → La–Cl exchange than for La–OH → La–F exchange. This explains the larger ΔV_th_(sat) when exchanging La-OH for fluoride than for chloride, cf. the discussion of the origin of threshold shift under ligand exchange in Section 3.1. The larger ΔV_th_(sat) for fluoride confers significant selectivity for fluoride vs. chloride, despite the limited difference in c_1/2_/K: In a WGTFT sensitised with La-loaded resin, a threshold shift of more than ~150 mV (i.e., more than ΔV_th_(sat) for chloride) can only be explained by the presence of fluoride. Using Equation (3) with the parameters from Table 1, to reach a threshold shift of 150 mV with fluoride we required a fluoride concentration of ~80 pM. This gave a more practical LoD than the 13 pM we determined with La-loaded resin in interferant-free deionised water: Beyond ~80 pM fluoride in water would give a threshold shift that cannot be due to chloride interferant. Still, this is 6 orders-of-magnitude below the fluoride potability limit of 79 µM. As a further interference test, we tested WGTFT response under the simultaneous presence of fluoride as analyte and chloride as interferant, which is more realistic than comparing response for analyte only vs. interferant only. Simultaneous testing allowed the checking of interactions between analyte and interferant from competition for binding sites. Figure 9 shows the response of WGTFTs with La-loaded chelating resin phase transfer membrane under samples containing both fluoride and chloride at equal concentration in the sample pool.

The response to simultaneous exposure to analyte (fluoride) and interferant (chloride) was similar to exposure to analyte alone (Figure 3), saturating at ΔV_th_(sat)~330 mV. The presence of interferant, therefore, did not pull ΔV_th_(sat) down from the level for fluoride alone, rather slightly increased it, but by far less than naive addition: ΔV_th_(sat) (F^−^ and Cl^−^) ≳ ΔV_th_(sat)(F^−^), but ΔV_th_(sat) (F^−^ and Cl^−^) < ΔV_th_(sat)(F^−^) + ΔV_th_(sat)(Cl^−^). At least qualitatively, the presence of fluoride is, therefore, still evident despite the simultaneous presence of interferant.

Interference from carbonate was more serious. c_1/2_ is similar to that of fluoride, and ΔV_th_(sat) exceeds 200 mV. ΔV_th_(sat) from carbonate is only slightly smaller to ΔV_th_(sat) for fluoride. Practically, the gap between ΔV_th_(sat) for analyte vs. interferant could be expanded by grinding the resin into a finer powder before membrane manufacture, as described in Section 3.2 Alternatively, carbonate removal prior to fluoride determination could be attempted (e.g., the ‘Gyrazur’^TM^ process [39] is used for carbonate removal in commercial water treatment works). However, we advise a more direct way to minimise carbonate (and other) interference in Section 3.4 below.

### 3.4. Reducing Carbonate Interference

**Figure 10:**
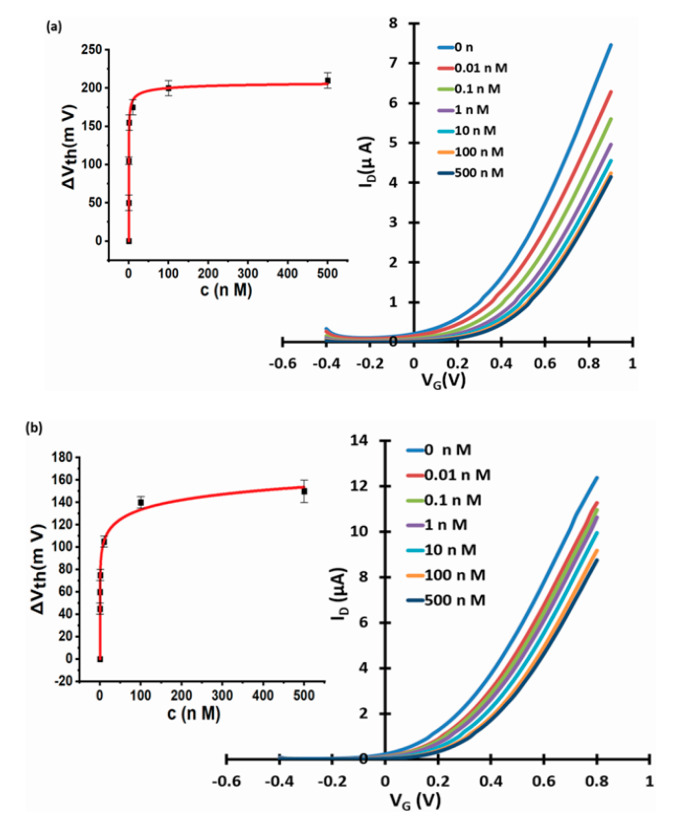
WGTFT fluoride sensors with lightly loaded resin under fluoride and carbonate. Response of lightly La-loaded resin to (**a**) fluoride and (**b**) carbonate. Insets: response characteristics with fits to Equation (3).

We repeated fluoride (analyte) and carbonate (interferant) sensing experiments with a phase transfer membrane filled with an only ‘lightly’ La-loaded resin. The preparation of lightly La-loaded resin by partial La extraction from a conventionally (heavily) loaded resin was described in Section 2.1. Figure 10a shows that a lightly La-loaded resin still strongly responds to fluoride. For response to carbonate (Figure 10b), we found a significantly reduced response for the lightly loaded resin than previously for the fully loaded resin (Figure 8c).

Parameters of response characteristics for the lightly loaded resin are summarised in Table 3 and compared to parameters from fully loaded resin.

We first note that ΔV_th_(sat) are very similar for lightly and fully loaded resin, which at first sight is somewhat surprising. However, we established in Section 3.1 that the characteristic concentration, c_1/2_, for threshold shift is more than 6 orders-of-magnitude smaller than c_1/2_ for fluoride extraction. Clearly, threshold shift saturates long before all available ion exchange sites have exchanged hydroxyl for fluoride. It appears that even the lightly loaded resin still has sufficient ion exchange sites to reach similar ΔV_th_(sat) as the fully loaded resin.

There are remarkable and useful differences in K, however, between the lightly and the fully loaded resins’ response to interferant carbonate: While K for the response to fluoride is not significantly reduced, K for carbonate response is at least 3 orders-of-magnitude smaller than for fully loaded resin. The stability constants of the equivalent La complexes favour ligand exchange for fluoride over carbonate: for the 1st La-F ligand binding, log K = 2.67 [40], but for the equivalent La-HCO_3_ interaction, lower log K = 1.40 was established [41]. While this is true in both fully and lightly loaded resin, the preference for fluoride over carbonate is masked in the fully loaded resin: We note that all sensors with Langmuir or LF characteristics can only select between analyte and interferant at low analyte concentrations, c, because fractional coverage θ(c) scales with K at low c only. At high concentrations (c >> 1/K) the LF law saturates (θ(c) → 1) for both analyte and interferant; hence, selectivity is lost. A similar saturation issue may occur under high concentration of sensitiser (ion exchange sites in the resin), rather than analyte/interferant: Despite lower K, all interferant will still be ion-exchanged when the concentration of ion exchange sites is too high. The results presented in this section show that the procedure described in Section 2.1 had the intended effect: On the one hand, response to interferant is far weaker, which is evidence for the removal of most La centres. On the other hand, response to analyte is still strong, which is evidence for some residual La centres. Further evidence for the presence of some residual La centres even after the removal procedure described in Section 2.1 comes from a control experiment shown in the Supplementary Section, Figure S5. This shows no response to fluoride for a membrane filled with as-received Puromet^TM^ MTS9501 resin that was never loaded with La. The resin in the membrane used for Figure 10, therefore, must have retained some La, otherwise it could not be responsive to fluoride. Overall, we find that diluting sensitiser opens a selectivity window that is closed at high sensitiser concentration.

For a full assessment of interference, further tests (e.g., sulfate, and nitrate) would have to be conducted to optimise the degree of La loading for best overall selectivity. While this is beyond the scope of this work, we establish diluting activated centres in an ion exchange resin as a general approach for improved selectivity.

## 4. Conclusions

We have provided a second example of an ion-exchanging rather than charge-accumulating sorbent as sensitiser in the phase transfer membrane of a water-gated field effect transistor (WGTFT) for potentiometric ion sensing. The fluoride-selective sorbent used here was derived by metal loading (La or Al) of a commercial macroporous aminophosphate (AMP) resin, ‘Puromet^TM^ MTS9501‘ to activate it for ion-exchange with fluoride. Despite being chemically very different from the previously used caesium-selective ion-exchanging crystalline zeolite mineral ‘mordenite’ [5] and despite anion vs. cation sorption, we found very similar response characteristics for both ion exchange media, which are distinctly different from the characteristics of charge-accumulating sensitisers (e.g., organic macrocycles, [1,2,3]): namely, that membrane potential, as revealed by WGTFT threshold shift, follows a Langmuir–Freundlich (LF) surface adsorption isotherm Equation (3) rather than a Nikolsky–Eisenman (modified Nernstian) law for charge accumulating ionophores. We also found that La-loaded resin is superior to Al-loaded resin. We assigned the membrane potential and consequential WGTFT threshold shift resulting from ion exchange to the different dipole moment of the exchanged (La–F) complex rather than original (La–OH) species, leading to a polarisation of the membrane. Moreover, grains ground to a finer powder led to larger saturated threshold shift at the same mass loading, suggesting a surface mechanism. The characteristic concentration, c_1/2_, for threshold shift is 6 orders-of-magnitude smaller than for mass uptake. The reason for this discrepancy is not obvious, but it enabled us to achieve a LoD that far undercuts practical requirements, which are sufficiently met by commercial solid-state membrane sensors. The most important practical limitation of our sensor concept for fluoride sensing is interference from co-solutes, particularly from carbonate. We have shown a successful strategy to combat interference from carbonate by reducing the degree of La loading of the ion exchange resin, which we believe can be generalised to other resin-based sensors with Langmuir or LF response characteristics.

Most importantly, with the example of fluoride, we established the general principle of extremely low LoD potentiometric sensing using organic ion exchange resins as sensitisers. LoD for fluoride far undercuts practical requirements, which are sufficiently met by the established electrochemical fluoride sensors with a Nikolsky–Eisenman (modified Nernstian) response. However, a number of similar ion exchange membranes are available as sorbents for highly toxic or precious trace elements in water, where extremely low LoD is essential and cannot be achieved by electrochemical sensors with Nikolsky–Eisenman characteristics. Examples are sorbent resins for arsenic- and gold-containing anions [20,21], and copper [22], mercury [23], and palladium [24] cations. Our work establishes a template for the way in which the entire organic resin sorbent family can be used as sensitisers in WGTFTs when an ultra-low limit-of-detection is essential.

## Figures and Tables

**Table 1:** Parameters of Langmuir–Freundlich fits to fluoride response. K, c_1/2_, ΔV_th_(sat), β, and limit of detection (LoD) for the response of WGTFTs sensitised with La-and Al-loaded chelating resins to fluoride. We only used coarse-ground resin-filled membranes. The results for fine-ground La-loaded resin from Figure 5 in Section 3.2 below are previewed here but discussed only later.

Metal Loading (Ground)	Fluoride Source	K [10^8^ L/mol]	c_1/2_ [pM]/[nM]	ΔV_th_(sat) [mV]	β	LoD [pM]
La (Coarse)	NaF	190 ± 80	(53 ± 22) pM	277 ± 14	0.43 ± 0.09	13
La (Fine)	NaF	114 ± 72	(88 ± 55) pM	541 ± 37	0.3 ± 0.04	0.82
La (Coarse)	KF	85 ± 39	(118 ± 54) pM	302 ± 16	0.3 ± 0.04	0.05
Al (Coarse)	NaF	3 ± 1.8	(3.3 ± 2) nM	137 ± 11	0.4 ± 0.12	600

**Table 2:** Parameters of Langmuir–Freundlich fits to interferant response. K, c_1/2_, ΔV_th_(sat), β, and LoD for the response of WGTFTs sensitised with La- and Al-/and La-loaded chelating resins to chloride/carbonate.

Metal Loading	Interferant	K [10^8^ L/mol]	c_1/2_ [pM/nM]	ΔV_th_(sat) [mV]	β	LoD [pM/nM]
La	Cl^−^ from NaCl	60 ± 40	720 pM *	152 ± 13	0.58 ± 0.2	0.5 nM
Al	Cl^−^ from NaCl	0.3 ± 0.7	28 nM *	112 ± 35	0.3 ± 0.1	1.1 nM
La	CO_3_^2−^ from Na_2_CO_3_	167 ± 56	(60 ± 34) pM	233 ± 9	0.5 ± 0.09	55 pM

* Due to the large error for K fitted by LF model, c_1/2_ is read graphically, directly from response characteristics.

**Table 3:** Parameters of Langmuir–Freundlich fits for lightly loaded vs. fully loaded resin. Parameters (K, c_1/2_, ΔV_th_(sat), β) for the response of WGTFTs sensitised with lightly La-loaded resin, extracted from Figure 10. For comparison, parameters for fully La-loaded resin are also shown (taken from Table 1 and Table 2).

	Fluoride		Carbonate	
Parameters ↓	Lightly Load	Fully Loaded	Lightly Loaded	Fully Loaded
K [10^6^ L/mol]	8500 ± 2100	19,000 ± 8000	4.4 ± 20	16,700 ± 5600
c_1/2_	(118 ± 29) pM	(53 ± 22) pM	234 nM	(60 ± 34) pM
ΔV_th_(sat) [mV]	211 ± 6	277 ± 14	288 ± 115	233 ± 9
β	0.43 ± 0.05	0.43 ± 0.09	0.2 ± 0.04	0.5 ± 0.09

## Supplementary Materials

The following are available online at https://www.mdpi.com/2072-666X/11/10/923/s1: **Figure S1**: Determination of limit- of- detection (LoD). B: Repeated of WGTFT fluoride (from NaF) response with La resin membrane. **Figure S3**: Repeated of WGTFT fluoride response with finely ground La resin membrane. **Figure S4**: Repeated of WGTFT fluoride (from NaF) response with Al resin membrane. **Table S1**: Parameters of Langmuir–Freundlich fits to fluoride response. **Figure S5**: Control experiment of WGTFT fluoride response with as- received Puromet MTS9501 resin. **Figure S6**: Determining the threshold voltage ΔVth (c) with its error for sensing F- with La resin- sensitised WGTFT.
