# Supplementary material for: Water-Gated Transistor Using Ion Exchange Resin for Potentiometric Fluoride Sensing
Source: Micromachines (Basel). 2020 Oct 5;11(10):923. doi: 10.3390/mi11100923 (PMC7601498; doi:10.3390/mi11100923)
Supplement: Supplementary file 1 [file micromachines-11-00923-s001.pdf]

Supplementary information

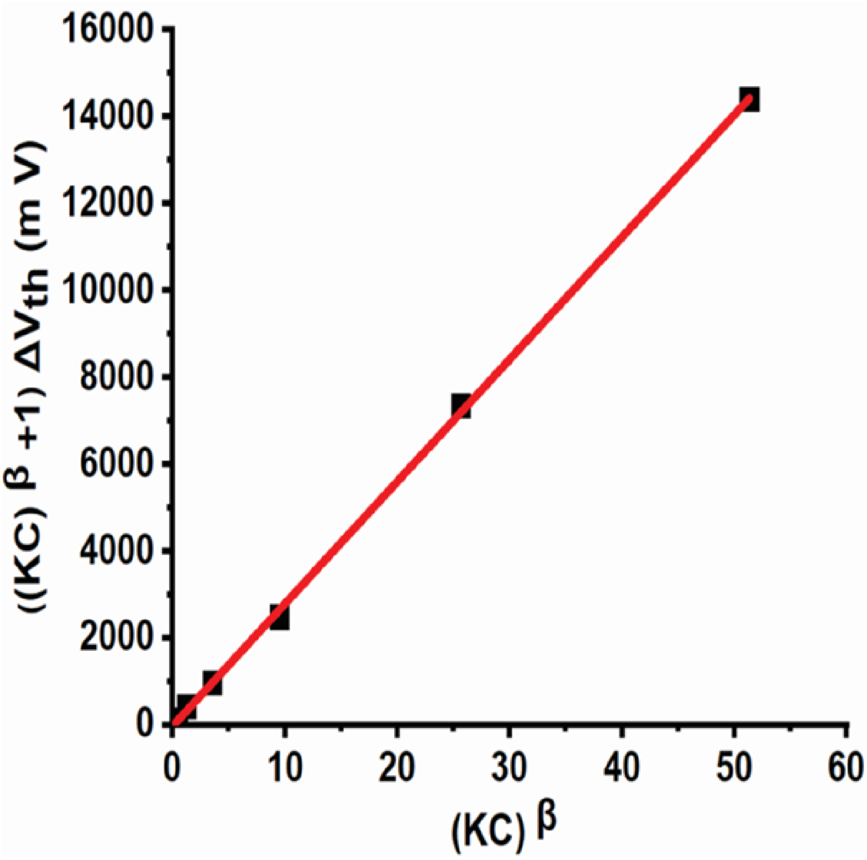

Fig. S1: Linearised response characteristics plot, ΔV_{th}(sat) $((Kc)^{\beta} + 1)$ *vs.* $(Kc)^{\beta}$, for the response characteristics, inset Fig. 2b, with straight- line- fits for determination of limit- of- detection (LoD) with eq. 4. All other LoDs reported here were determined by similar plots and analysis.

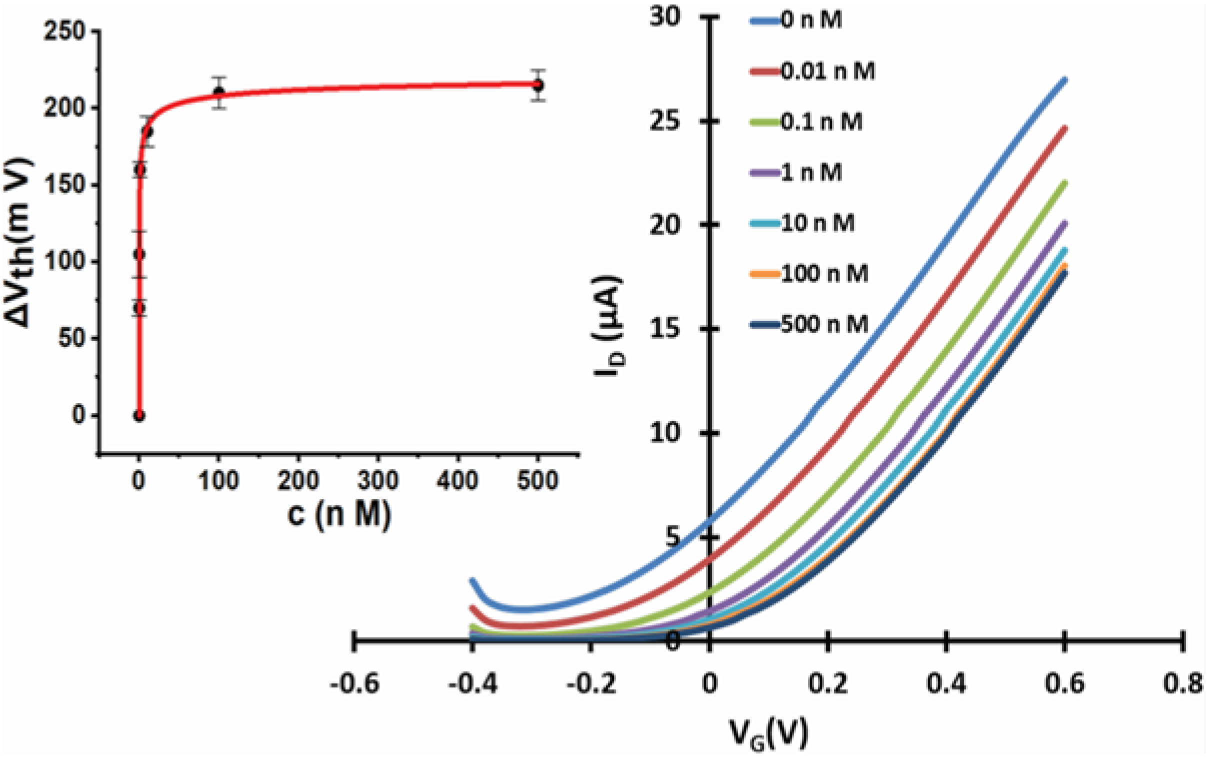

Fig. S2: Repeated transfer characteristics of La loaded chelating resin- sensitised SnO_2 WGTFT gated under increasing F^- concentrations from NaF in the outer pool. Inset: Response characteristic with fit to eq. 3.

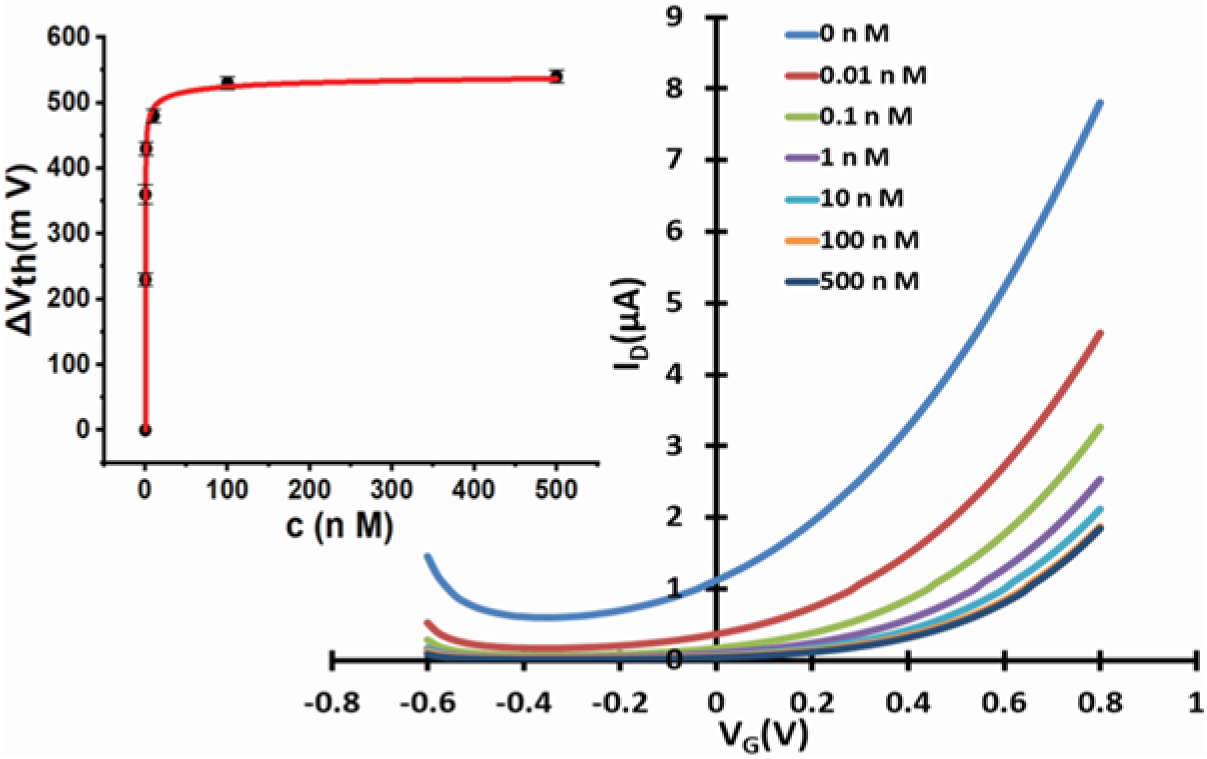

Fig. S3: Repeated transfer characteristics of finer ground La loaded chelating resin-sensitised SnO_2 WGTFT gated under increasing F^- concentrations from NaF in the outer pool. Inset: Response characteristic with fit to eq. 3.

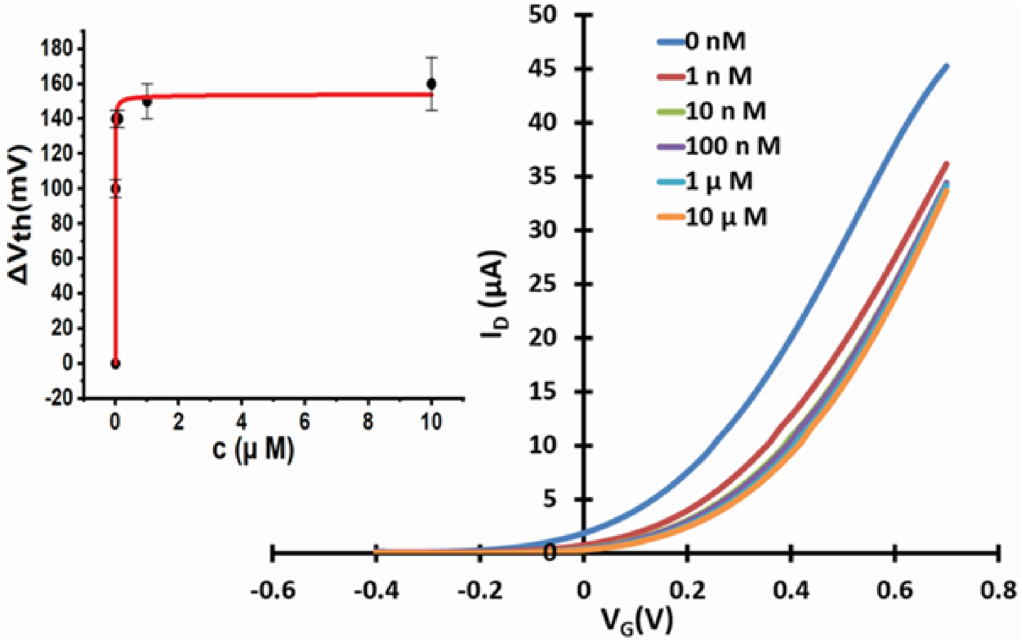

Fig. S4: Repeated transfer characteristics of Al loaded chelating resin- sensitised SnO_2 WGTFT gated under increasing F^- concentrations from NaF in the outer pool. Inset: Response characteristic with fit to eq. 3.

Table S1: K, $c_{1/2}$, ΔV_{th}(sat), β, and LoD for the repeated response of WGTFTs sensitised with La- and Al loaded chelating resins to fluoride corresponding to table 1 in the main manuscript. These parameters are similar to their values in table 1 within error.

Metal loading (ground)	Fluoride source	K [10^8 L/mol]	$c_{1/2}$ [pM] / [nM]	ΔV_{th}(sat) [mV]	β	LoD [pM]
La (coarse)	NaF	85 ±21	(118±30) pM	227 ± 7	0.35 ±0.03	0.25
La (fine)	NaF	432±95	(23 ±5)pM	555±14	0.34±0.04	0.13
Al (coarse)	NaF	33±20	(0.3± 0.18) nM	154±6	0.55±0.2	73

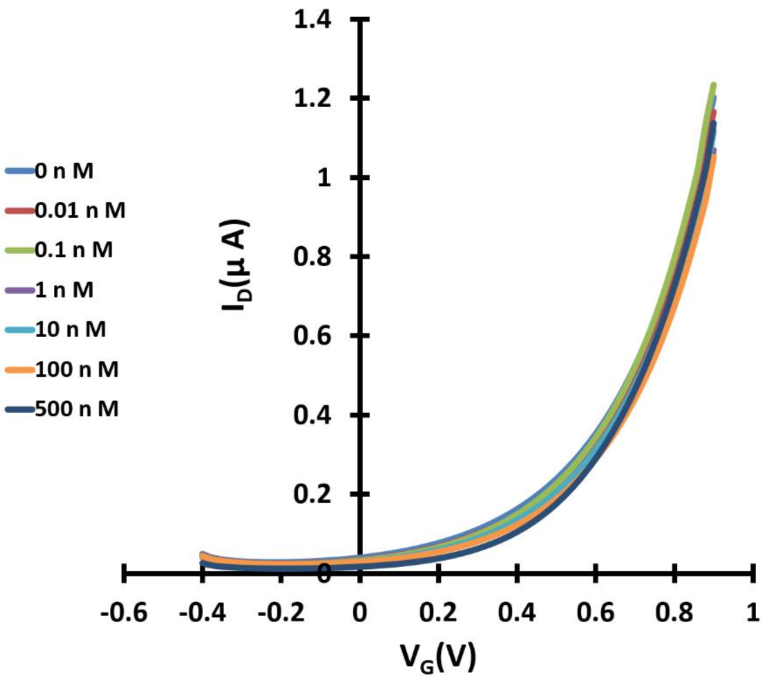

Figure S5. Control experiment: WGTFT using a membrane loaded with as- received Puromet MTS9501 resin that was never activated with La. In the absence of La, no response to fluoride is observed.

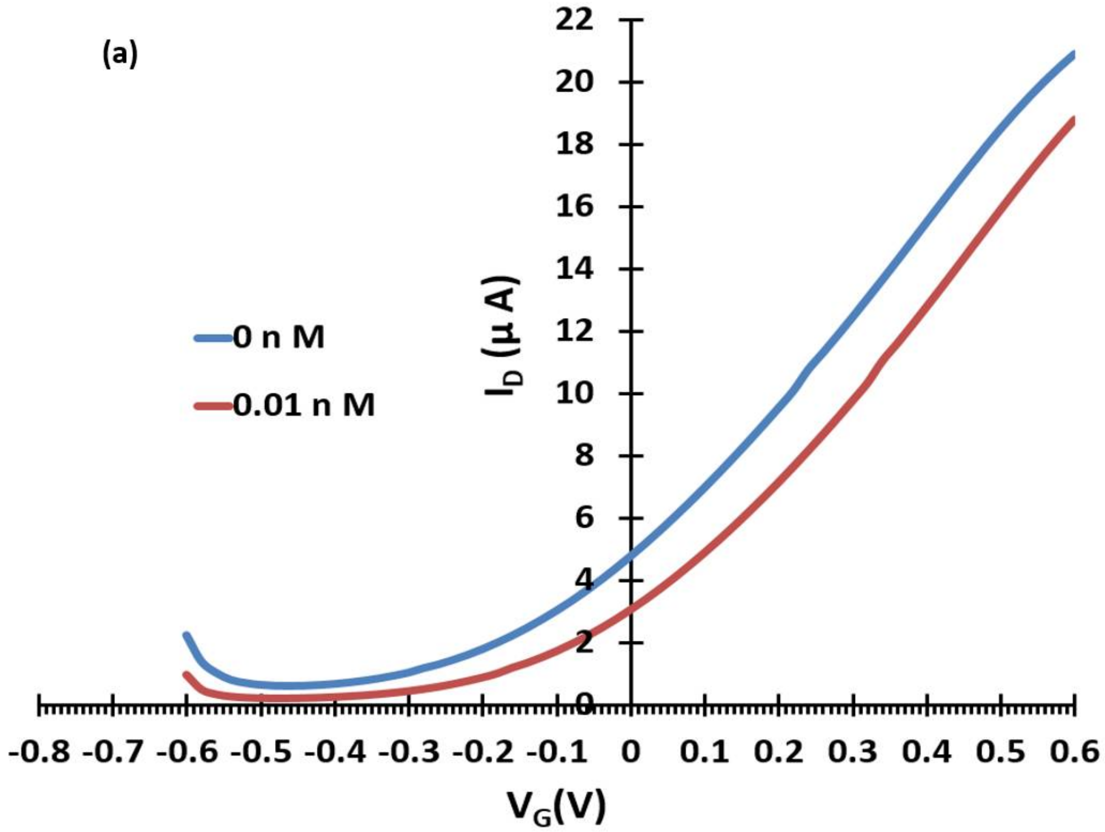

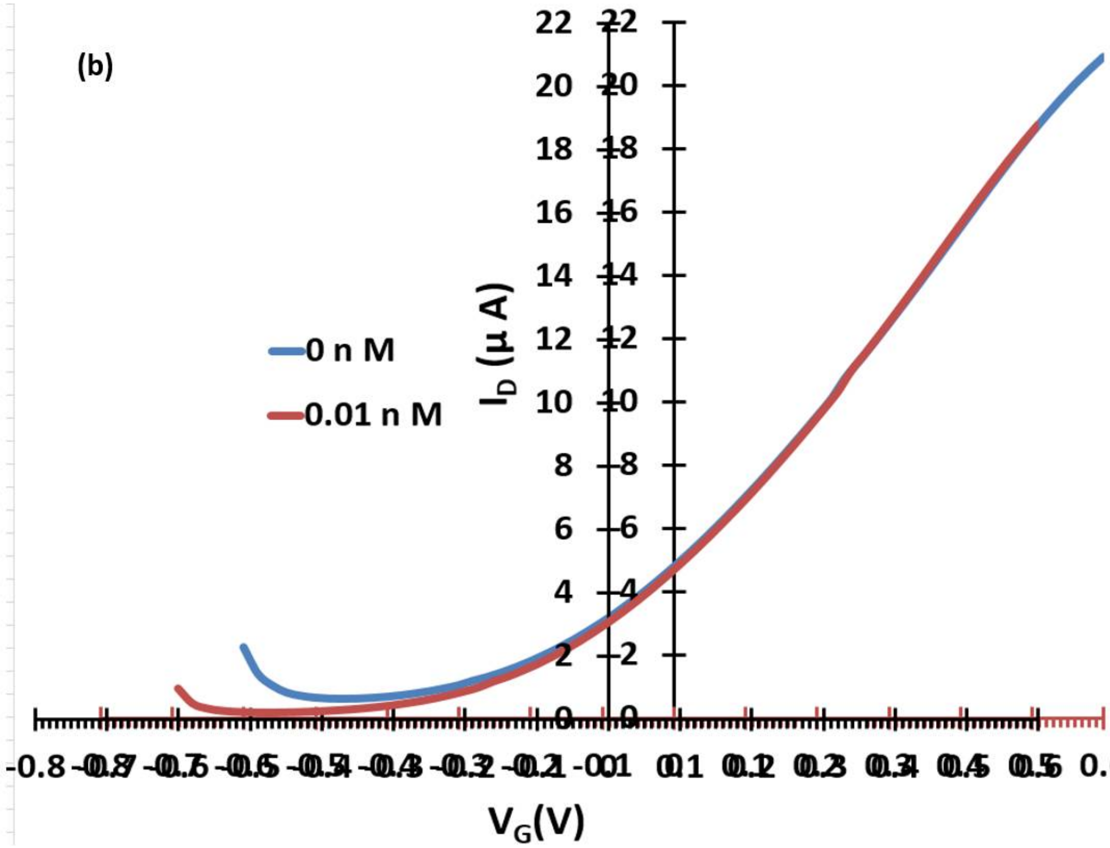
(b)
0 n M
0.01 n M
I_D (µ A)
V_G(V)

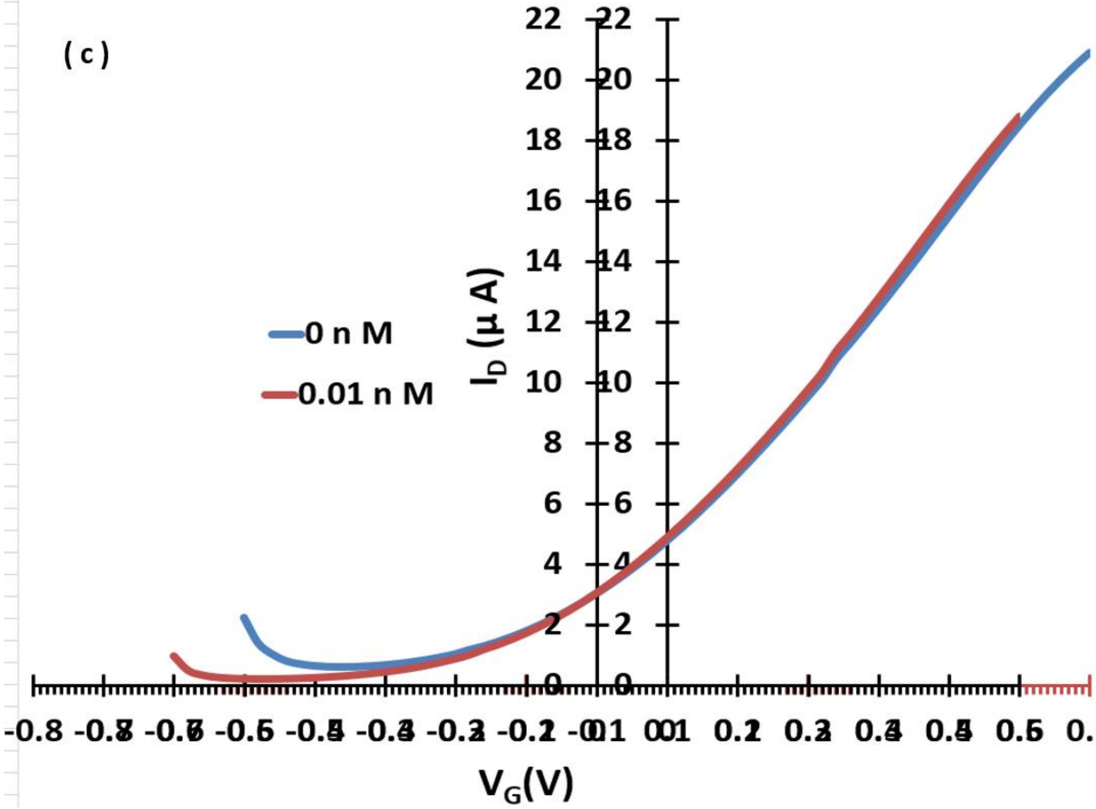
(c)
0 n M
0.01 n M
I_D (µ A)
V_G(V)

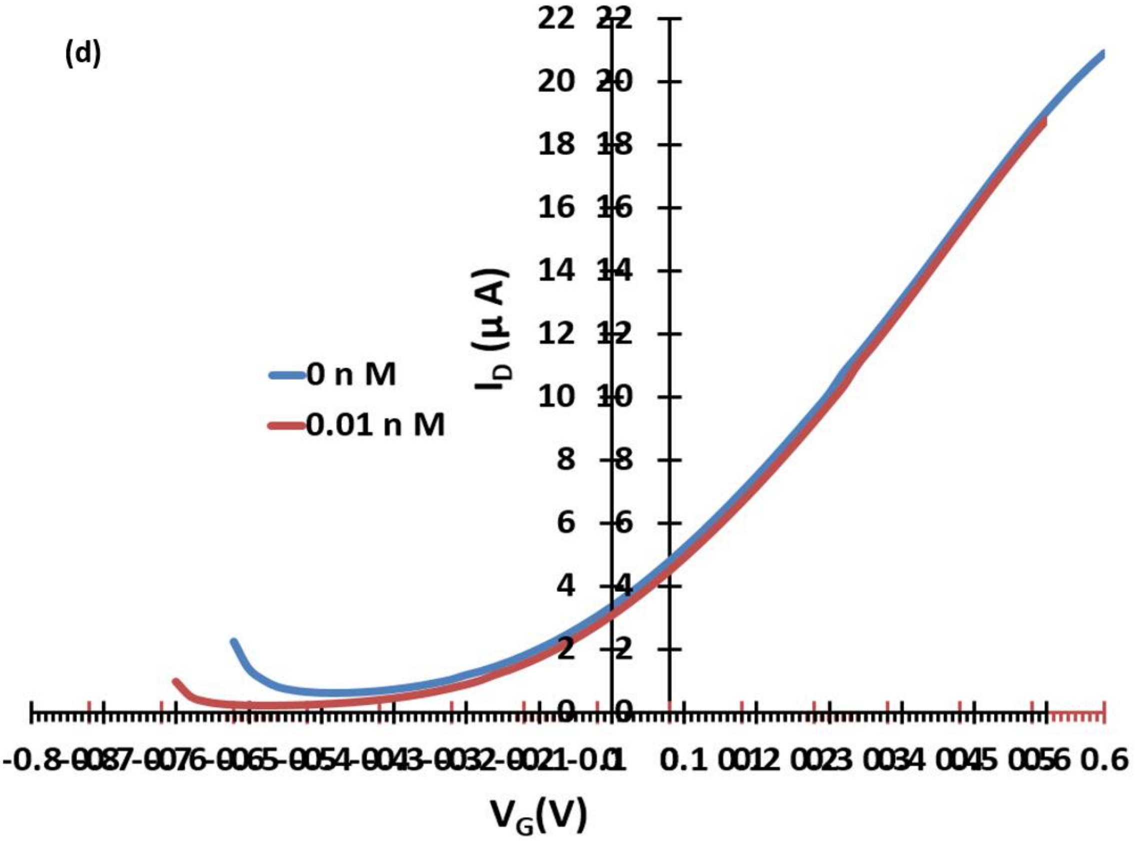

Figure S6. These figures illustrate an example for determining the threshold voltage ΔV_{th} (c) with its error for sensing F^- (from NaF) with La-loaded chelating resin- sensitised SnO_2 WGTFT platform. The transfer characteristics for c = 0 and 0.01 nM fluoride concentration are shown in Figure S6a. Figure S6b shows the 0.01 nM fluoride transfer characteristic shifted along the gate voltage (V_G) axis by 90 mV for visually best match to the c = 0 fluoride transfer. Shifting by 5mV more (Figure S6c) or less (Figure S6d) already gives a visible mismatch. Hence, in this example, ΔV_{th} (0.01 nM F^-) = (90 +/- 5) mV.
